# Prolonged Action Potential and After depolarizations Are Not due to Changes in Potassium Currents in NOS3 Knockout Ventricular Myocytes

**DOI:** 10.1155/2012/645721

**Published:** 2012-08-28

**Authors:** Honglan Wang, Ingrid M. Bonilla, Xin Huang, Quanhua He, Mark J. Kohr, Cynthia A. Carnes, Mark T. Ziolo

**Affiliations:** ^1^Department of Physiology and Cell Biology, The Dorothy M. Davis Heart and Lung Research Institute, The Ohio State University, Columbus, OH 43221, USA; ^2^College of Pharmacy, The Dorothy M. Davis Heart and Lung Research Institute, The Ohio State University, Columbus, OH 43210, USA; ^3^Translational Medicine Branch, National Heart, Lung, and Blood Institute, NIH, Bethesda, MD 20892, USA

## Abstract

Ventricular myocytes deficient in endothelial nitric oxide synthase (NOS3^−/−^) exhibit prolonged action potential (AP) duration and enhanced spontaneous activity (early and delayed afterdepolarizations) during **β**-adrenergic (**β**-AR) stimulation. Studies have shown that nitric oxide is able to regulate various K^+^ channels. Our objective was to examine if NOS3^−/−^ myocytes had altered K^+^ currents. APs, transient outward (*I*_to_), sustained (*I*_Ksus_), and inward rectifier (*I*_K1_) K^+^ currents were measured in NOS3^−/−^ and wild-type (WT) myocytes. During **β**-AR stimulation, AP duration (measured as 90% repolarization-APD_90_) was prolonged in NOS3^−/−^ compared to WT myocytes. Nevertheless, we did not observe differences in *I*_to_, *I*_Ksus_, or *I*_K1_ between WT and NOS3^−/−^ myocytes. Our previous work showed that NOS3^−/−^ myocytes had a greater Ca^2+^ influx via L-type Ca^2+^ channels with **β**-AR stimulation. Thus, we measured **β**-AR-stimulated SR Ca^2+^ load and found a greater increase in NOS3^−/−^ versus WT myocytes. Hence, our data suggest that the prolonged AP in NOS3^−/−^ myocytes is not due to changes in *I*_to_, *I*_Ksus_, or *I*_K1_. Furthermore, the increase in spontaneous activity in NOS3^−/−^ myocytes may be due to a greater increase in SR Ca^2+^ load. This may have important implications for heart failure patients, where arrhythmias are increased and NOS3 expression is decreased.

## 1. Introduction

Cardiac myocytes endogenously produce nitric oxide (NO) via two constitutively expressed NO synthase isoforms: endothelial NO synthase (NOS3) and neuronal NO synthase (NOS1). Both NOS1 and NOS3 play important roles in modulating cardiac function [1]. However, NOS1 and NOS3 signalings lead to different functional effects [2, 3]. NOS1 is localized to the sarcoplasmic reticulum (SR) and enhances cardiac contraction [2, 4], while NOS3 is localized to the caveolae and blunts the response to *β*-adrenergic (*β*-AR) stimulation due to a decreased L-type Ca^2+^ current (*I*_Ca_) [5, 6]. We also observed that NOS3 knockout (NOS3^−/−^) myocytes have prolonged action potential (AP) duration [5]. 

In addition to Ca^2+^ channels in the venitrcular myocyte, the AP waveform is also determined by potassium (K^+^) channels, which are essential for proper electrical activity of the heart as they are responsible for the resting membrane potential (RMP), the plateau phase, and repolarization [7]. K^+^ channels are also the most variable channels with multiple components, such as transient outward K^+^ current (*I*_to_), sustained outward K currents (*I*_Ksus_), inward rectifier K^+^ current (*I*_K1_), delayed rectifier K^+^ current (*I*_Ks_), and so forth [7]. In addition, there are different expression patterns of the various K^+^ channels in different species [8].

A previous study has shown that sodium nitroprusside, a NO donor, can enhance *I*_Ks_ in guinea-pig cardiac myocytes [9]. Another study found that NOS3, but not NOS1, is responsible for the Ca^2+^-induced *I*_Ks_ enhancement [10]. *I*_Ks_, which activates very slowly during depolarization and deactivates very slowly during repolarization, contributes to the repolarization during the late phase of the AP. However, *I*_Ks_ channels are not expressed in adult murine ventricular myocytes [8, 11]. Thus, our observed changes in AP waveform in NOS3^−/−^ mouse myocytes may result from alterations of other K^+^ channels, such as *I*_Ksus_, *I*_to_, and/or *I*_K1_. In mouse hearts, *I*_Ksus_ is an important modulator of the plateau phase and repolarizing phase 3 of the AP. *I*_to_ contributes to the repolarizing phase 1 and the plateau phase of AP. Previous data [12] showed that NO and NO donors inhibited human atrial *I*_to_. *I*_K1_ is responsible for setting the RMP and shaping the late repolarizing phase 3 of the AP. Gómez et al. [13] found that NO and NO donors can increase *I*_K1_ measured in human atrial cells. However, the role of NOS3 signaling on modulation of *I*_to_, *I*_Ksus_, and/or *I*_K1_ in ventricular myocytes is unclear. In addition to contributing to prolonged AP duration, altered K^+^ channel function can contribute to the generation of arrhythmias [14, 15]. Indeed, there is an increase in the incidence of arrhythmias in NOS3^−/−^ mice [16]. Furthermore, our and others' data have shown increased spontaneous activity (i.e., early and delayed afterdepolarizations) in NOS3^−/−^ myocytes [5, 17]. Thus, the purpose of this study is to determine if NOS3 signaling modulates K^+^ currents. In order to investigate the effects of NOS3 signaling on K^+^ currents, we used ventricular myocytes isolated from NOS3^−/−^ and corresponding WT mice to investigate if alterations in one or all of K^+^ channels (*I*_to_, *I*_Ksus_, *I*_K1_) play a role in the prolongation of the AP, an effect expected to increase the propensity for spontaneous activity in NOS3^−/−^ myocytes.

## 2. Materials and Methods

### 2.1. Isolation of Ventricular Myocytes

Age-matched, male and female NOS3^−/−^ mice [18] and their corresponding control (C57Bl/6J) were obtained from Jackson Laboratories (Bar Harbor, ME, USA). Ventricular myocytes were isolated as previously described [5]. Briefly, the heart was mounted on a Langendorff apparatus and perfused with Ca^2+^ free normal Tyrode solution for 4 min. Blendzyme Type IV (0.077 mg/mL) (Roche Applied Science, Indianapolis, IN, USA) was then added to the perfusate. After 2–5 minutes, the heart was taken down, the right and left ventricles minced, and myocytes dissociated by trituration. Subsequently the myocytes were filtered, centrifuged, and resuspended in normal Tyrode solution containing 200 *μ*mol/L Ca^2+^. We randomly selected our myocytes and the region of the heart (epicardium, endocardium, midmyocardium, etc.) could not be determined. Myocytes were used within 6 hours of isolation. All the animal protocols and procedures were performed in accordance with National Institutes of Health guidelines and approved by the Institutional Laboratory Animal Care and Use Committee at The Ohio State University.

### 2.2. Measurement of Action Potential

Action potentials (AP) were recorded with an Axopatch-200B amplifier and pClamp 8.1 software (Axon Instrument, Foster City, CA, USA) using the current-clamp mode. The pipette was filled with (in mM): NaCl (8), KCl (10), K-Aspartate (140), HEPES (5), MgATP (2), and pH 7.2 adjusted with KOH or HCl, with a resistance of 9–11 MΩ. Myocytes were perfused with normal Tyrode solution, which consisted of (in mM): NaCl (140), KCl (4), MgCl_2_ (1), CaCl_2_ (1), Glucose (10), HEPES (5), L-arginine (1), and pH 7.4 adjusted with NaOH or HCl. Isoproterenol (ISO, 1 *μ*M, Sigma), a nonselective *β*-AR agonist, was prepared fresh each experimental day. A Grass S48 stimulator gated the amplifier for current injection to activate the AP, triggered by a 1.5 ms, 2 nA current injection. Measurements were performed at 37 ± 1°C.

### 2.3. Measurement of Potassium Currents

The amphotericin-B-perforated patch clamp technique was used to measure the various K^+^ currents. Myocytes were placed in a laminin-coated cell chamber (Cell Microcontrols, Norfolk, VA, USA) and superfused with bath solution containing (in mM): 135 NaCl, 5 MgCl_2_, 5 KCl, 10 glucose, 1.8 CaCl_2_, and 5 HEPES with pH adjusted to 7.4 with NaOH at temperature of 36 ± 0.5°C. Nifedipine (2 *μ*M) was added to the superfusate to block the L-type calcium current. Borosilicate glass micropipettes with tip resistance of 1.5–3 MΩ were filled with pipette solution containing the following (in mM): 100 K-aspartate, 40 KCl, 5 MgCl, 5 EGTA, 5 HEPES, and pH adjusted to 7.2 with KOH.

Inward rectifier K^+^ current (*I*_K1_) was elicited by voltage steps from −140 to +40 mV from a holding potential of −40 mV (which will inactivate the sodium current). The current was measured at the end of each 100-ms test pulse. *I*_K1_ inward conductance (mS/cm_2_) was determined by calculating the slope of the linear portion of the current density-voltage relationship from −140 to −100 mV. Peak outward *I*_K1_ density was measured as the current at −60 mV (*I*_−60_) [19]. All currents (in picoamperes (pA)) were normalized to the cell capacitance (measured in picofarads (pF)) and expressed as pA/pF.

Outward K^+^ currents were elicited by a series of 300 ms test potentials from −50 to +50 mV from a holding potential of −60 mV (which will inactivate the sodium current). The sustained K^+^ current (*I*_Ksus_) was measured at the end of the 300 ms test pulse. The transient outward K^+^ current (*I*_to_) was determined by subtracting the sustained outward current from the peak outward current [19].

### 2.4. SR Ca^2+^ Load

 SR Ca^2+^ load was measured at room temperature (22°C) by rapid application of 10 mmol/L caffeine for 10 sec. The amplitude of the caffeine-induced Ca^2+^ transient was used as an index of SR Ca^2+^ load [20].

### 2.5. Statistics

Myocyte data were presented as mean ± SEM. Differences between multiple groups were evaluated for statistical significance using an ANOVA (followed by Neuman-Keuls test) or unpaired Student's *t*-test for two groups. Statistical significance was accepted at the level of *P* < 0.05.

## 3. Results

### 3.1. NOS3^−/−^ Myocytes Have Prolonged *β*-AR Stimulated APD but No Change in RMP

**Figure 1:**
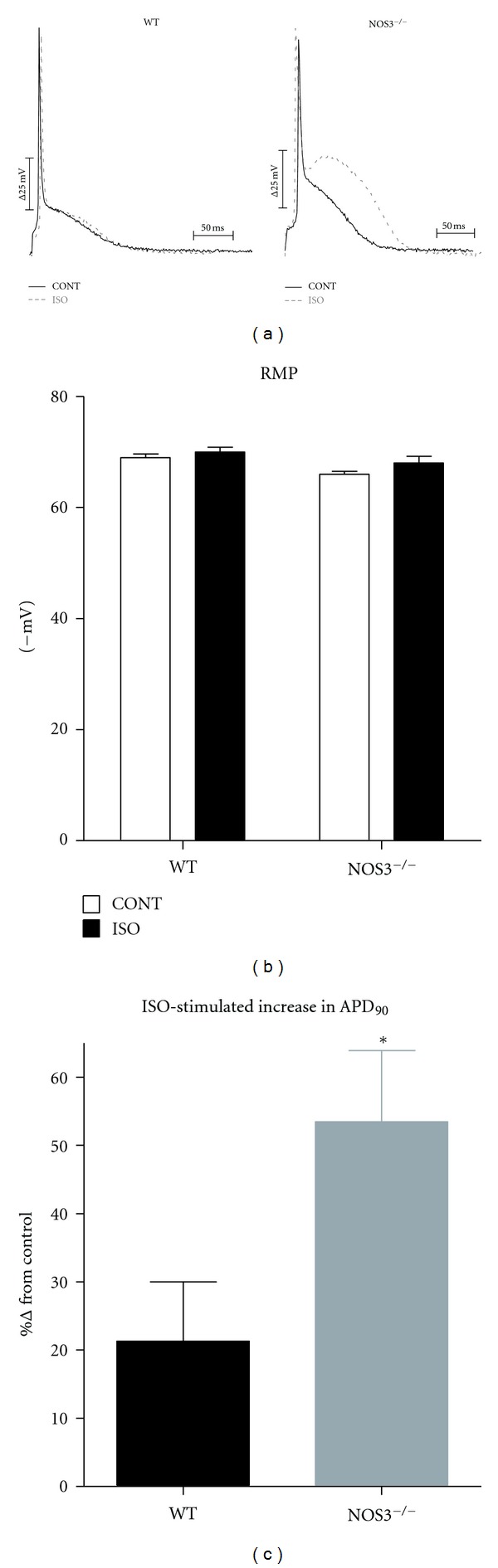
Prolonged APD90 in NOS3^−/−^ myocytes after *β*-AR stimulation. (a) Representative AP traces from WT and NOS3^−/−^ myocytes (control-CONT; isoproterenol-ISO). (b) Summary data (mean ± S.E.M.) of RMP in WT and NOS3^−/−^ myocytes. (c) Summary data (mean ± S.E.M.) of *β*-AR stimulated increase of APD90 in WT and NOS3^−/−^ myocytes. *P < 0.05, *n* = 4, 7 cells.

 We have previously shown that during *β*-AR stimulation, NOS3^−/−^ ventricular myocytes had a prolonged AP (measured as time to 90% repolarization-APD_90_) when measured at room temperature [5]. We repeated these experiments to examine if the phenomenon occurred at body temperature. Thus, we measured AP waveform in control (wildtype, WT) and NOS3^−/−^ myocytes at 37°C. Representative AP traces measured in a WT and NOS3^−/−^ myocyte (±*β*-AR stimulation with ISO) are shown in Figure 1(a). As shown in Figure 1(b), there was no difference between WT and NOS3^−/−^ resting membrane potential (basal or with *β*-AR stimulation). However, we found that during *β*-AR stimulation, NOS3^−/−^ myocytes (compared to WT) had a significant increase in APD_90_, consistent with our previous finding (Basal, WT: 75 ± 17 ms, NOS3^−/−^: 77 ± 10 ms; ISO, WT: 93 ± 24 ms, NOS3^−/−^: 117 ± 17 ms). The increase in APD_90_ with *β*-AR stimulation in NOS3^−/−^ myocytes is shown in Figure 1(c). These data suggest that NOS3^−/−^ myocytes have a prolonged AP with no change in the RMP during *β*-AR stimulation.

### 3.2. NOS3^−/−^ Myocytes Do Not Have Altered Inward Rectifier K^+^ Currents

**Figure 2:**
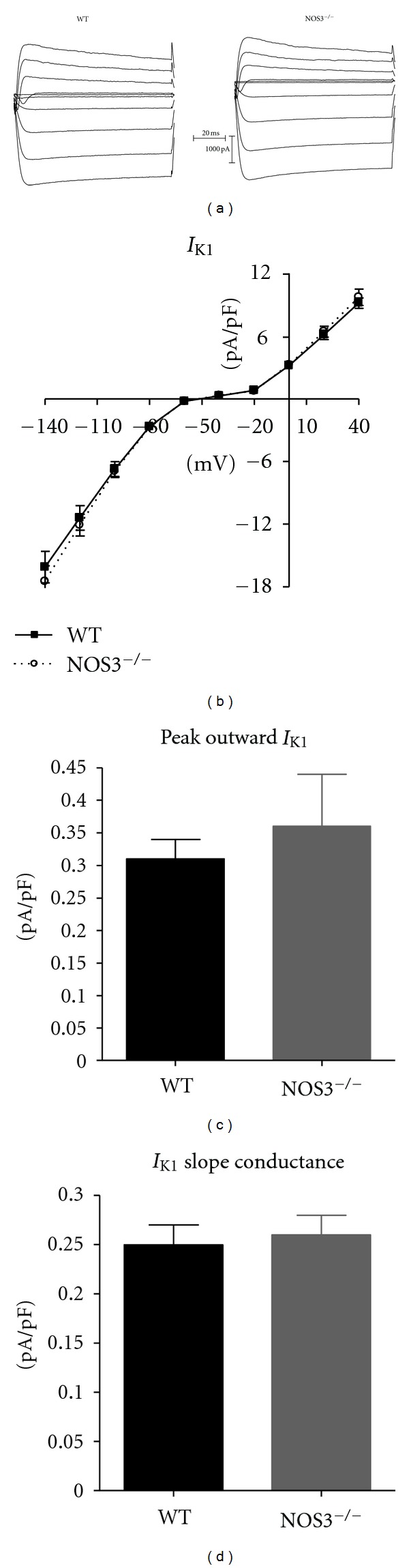
*I*_K1_ current was not changed in NOS3^−/−^ myocytes. (a) Representative traces from WT and NOS3^−/−^ myocytes. (b) *I-V* curves of *I*_K1_ in WT and NOS3^−/−^ myocytes. (c) Summary data (mean ± S.E.M.) of peak outward *I*_K1_ in WT and NOS3^−/−^ myocytes. (d) Summary data (mean ± S.E.M.) of *I*_K1_ slope conductance in WT and NOS3^−/−^ myocytes. *n* = 6, 15 cells.

 An important determinant of RMP and APD_90_ is the inward rectifier K^+^ current (*I*_K1_). Since we had prolonged APD_90_ with *β*-AR stimulation in NOS3^−/−^ myocytes, we investigated if these myocytes had altered *I*_K1_. Representative *I*_K1_ currents measured in a WT and NOS3^−/−^ myocyte are shown in Figure 2(a). As shown in Figures 2(b)–2(d), knockout of NOS3 did not alter the *I*_K1_ *I*-*V* relationship, the peak outward *I*_K1_, or the *I*_K1_ slope conductance. These data suggest that NOS3 signaling does not modulate *I*_K1_.

### 3.3. NOS3^−/−^ Myocytes Do Not Have Altered Transient Outward or Sustained K^+^ Currents

**Figure 3:**
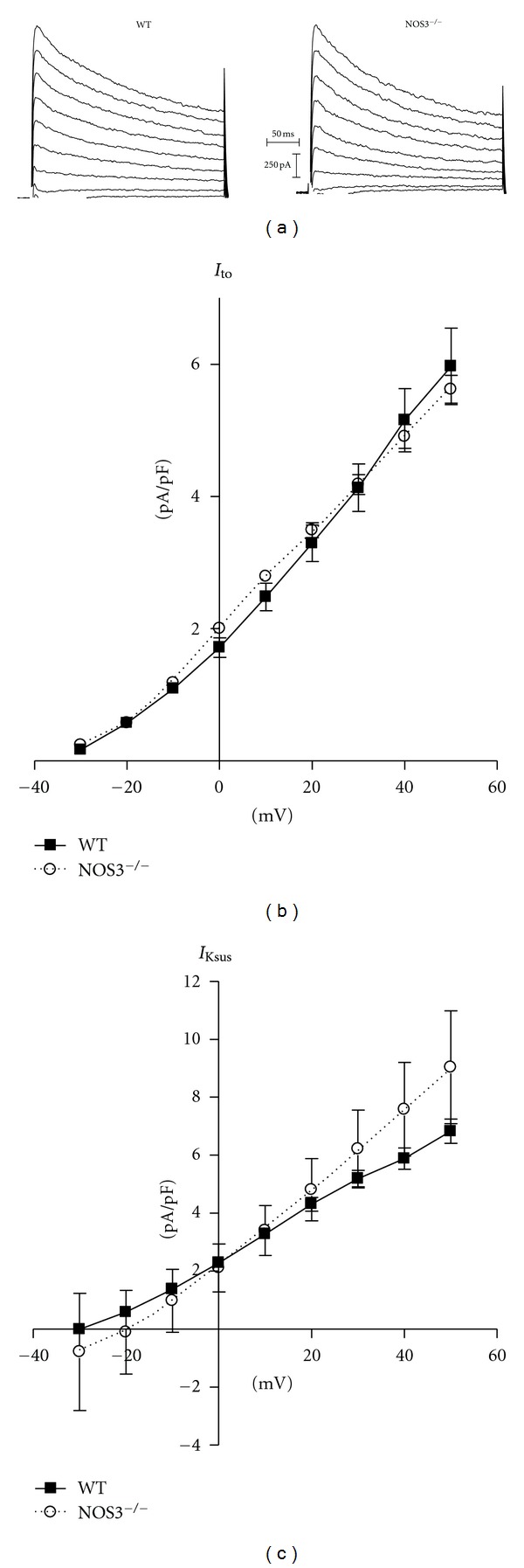
NOS3 knockout did not alter *I*_to_ or *I*_Ksus_. (a) Representative current traces from WT and NOS3^−/−^ myocytes. (b) Summary data (mean ± S.E.M.) of *I*_to_ *I-V* curves in WT and NOS3^−/−^ myocytes. (c) Summary data (mean ± S.E.M.) of *I*_Ksus_ *I-V* curves in WT and NOS3^−/−^ myocytes. *n* = 7, 13 cells.

 Since other K^+^ currents are also involved in repolarization to determine the APD, we investigated if NOS3^−/−^ myocytes had alterations in other K^+^ currents by measuring *I*_to_ and *I*_Ksus_. Representative currents measured in a WT and NOS3^−/−^ myocyte are shown in Figure 3(a). Our data show no difference in the *I*_to_ or *I*_Ksus_ *I-V* relationship in NOS3^−/−^ compared to WT myocytes (Figure 3). With our voltage protocol, *I*_Ksus_ is composed of various K^+^ currents, including *I*_K,slow_. However, since we did not observe a difference in composite *I*_Ksus_, we did not further investigate the contributing currents. These data indicate that NOS3 signaling does not modulate *I*_to_ or *I*_Ksus_.

### 3.4. *β*-AR Stimulated SR-Ca^2+^ Load Was Higher in NOS3^−/−^ Myocytes

**Figure 4:**
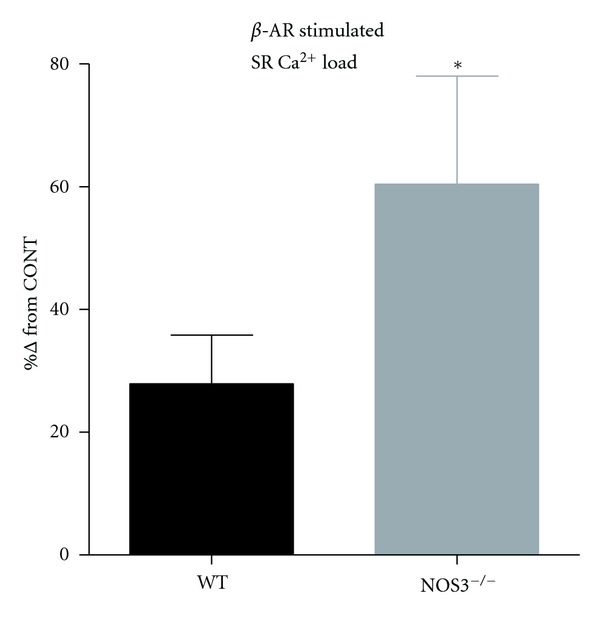
Larger increase in *β*-AR stimulated SR Ca^2+^ load in NOS3^−/−^ myocytes. Summary data (mean ± S.E.M.) of the increase in SR Ca^2+^ load with *β*-AR stimulation in WT and NOS3^−/−^ myocytes (control- CONT). *P < 0.05 versus WT, *n* = 11, 27 cells.

 Altered K^+^ currents resulting in prolonged APD can contribute to the generation of afterdepolarizations [21, 22]. However, since we did not observe any differences in *I*_to_, *I*_Ksus_, or *I*_K1_, we performed additional experiments to measure SR Ca^2+^ load, which is also known to contribute to afterdepolarizations [22, 23]. We observed no difference in basal SR Ca^2+^ loads between NOS3^−/−^ and WT myocytes (1.8 ± 0.2 versus 1.9 ± 0.2 Δ*F/F *_0_). However, our data (Figure 4) show that NOS3^−/−^ myocytes (compared to WT) have a larger increase in their SR Ca^2+^ load with *β*-AR stimulation. These data suggest that with *β*-AR stimulation there is a larger increase in SR Ca^2+^ load in NOS3^−/−^ myocytes.

## 4. Discussion

 The purpose of this study was to investigate the role of NOS3 signaling on the modulation of *I*_to_, *I*_Ksus_, and *I*_K1_. Our data show that *I*_to_, *I*_Ksus_, and *I*_K1_ are not altered in NOS3^−/−^ ventricular myocytes, which suggests that NOS3 does not modulate *I*_to_, *I*_Ksus_, and *I*_K1_. Furthermore, the increased afterdepolarizations with *β*-AR stimulation in NOS3^−/−^ myocytes may be due to enhanced spontaneous Ca^2+^ waves resulting from increased *β*-AR stimulated SR Ca^2+^ load.

### 4.1. Nitric Oxide Modulation of the *β*-AR Pathway

 Stimulation of *β*-AR pathway is an essential regulator of cardiac contractility, leading to positive inotropic and lusitropic effects [24]. Our and other previous studies showed that NOS3 signaling blunts the functional response to *β*-AR stimulation [2, 5, 25–28]. That is, NOS3^−/−^ myocytes have increased Ca^2+^ transient amplitudes and shortening amplitudes compared to WT, which is due to an enhanced *β*-AR stimulated *I*_Ca_. Our data also demonstrated that NOS3^−/−^ myocytes had prolonged APD during *β*-AR stimulation [5]. This current study examined if alterations in K^+^ currents contributed to the prolonged APD in NOS3^−/−^ myocytes.

### 4.2. Prolonged AP in NOS3^−/−^ Myocytes Is Not due to Altered *I*_to_, *I*_Ksus_, and *I*_K1_

Previous studies have shown that alterations in *I*_K1_, which is an important K^+^ channel responsible for setting the RMP and shaping the late phase 3 of AP, can contribute to prolonging the AP [14]. However, our data (Figure 2) shows that *I*_K1_ is unaffected with knockout of NOS3. Furthermore, we observed similar RMP between WT and NOS3^−/−^ myocytes (Figure 1), consistent with no difference in *I*_K1_. Thus, *I*_K1_ is not the cause for prolonged APD in NOS3^−/−^ myocytes.

 Besides *I*_K1_, many other K^+^ currents are key channels in shaping the AP waveform. *I*_to_ is a transient outward current that contributes to the plateau phase of AP. Inhibition of *I*_to_ has the ability to prolong the APD. In mouse myocytes, *I*_Ksus_ is the sustained outward component of the repolarizing K^+^ current, and it plays an important role during the plateau and phase 3 of the AP. Interestingly, we also did not observe any differences in *I*_to_ and *I*_Ksus_ between NOS3^−/−^ and WT myocytes (Figure 3). These data indicate that *I*_to_ or *I*_Ksus_ is not involved in the prolonged APD in NOS3^−/−^ myocytes. Using our voltage protocol to elicit outward K^+^ channels, our composite *I*_Ksus_ current will encompass *I*_K,slow_ [29–32]. *I*_K,slow_ is also an important K^+^ current in mice responsible for ~30% of the repolarization [29]. Since we did not observe a difference in *I*_Ksus_, these data suggest that *I*_K,slow_ is also not modulated by NOS3 signaling. It should be noted that *I*_to_ and *I*_Ksus_ (including *I*_K,slow_) are not the only operating currents during the plateau phase. *I*_Ca_ is an inward Ca^2+^ current that is counterbalanced by *I*_to_ and *I*_Ksus_, which creates the plateau phase of the AP. Hence, the duration of the plateau is determined by the balance between *I*_Ca_ and *I*_to_ and *I*_Ksus_. We previously observed an increase in the *β*-AR stimulated *I*_Ca_ in NOS3^−/−^ myocytes compared to WT myocytes [5]. Thus, we suggest that in NOS3^−/−^ myocytes an increased *I*_Ca_ will overcome the unaltered *I*_to_ and *I*_Ksus_, leading to more inward current resulting in prolonging the APD. This is consistent with previous studies which demonstrated that changes in *I*_Ca_ will affect APD [33–35].

Previous studies demonstrated that NO donors are able to modulate K^+^ currents. The discrepancy between our findings and other groups may be due to the use of different species (human, guinea pig, and mouse). Another distinction between our current study and the previous studies is the use of endogenous NO (i.e., NOS3) or exogenous NO (i.e., NO donors). Our and others', work has shown the endogenous NO signaling via NOS1 and NOS3 is compartmentalized [1, 2]. However, when using NO donors, the entire myocyte will be exposed to NO, which is in contrast to the localized signaling observed with endogenous NO. In this study, we used isolated myocytes from NOS3^−/−^ and WT hearts to directly investigate the effects of NOS3 signaling on K^+^ currents. While Gómez et al. [12] did observe effects of NO donors on *I*_to_, they also did not observe any differences in *I*_to_ between WT and NOS3^−/−^ ventricular myocytes (consistent with our observations). Thus, we are confident in our findings that NOS3 signaling does not modulate *I*_to_, *I*_Ksus_, or *I*_K1_.

### 4.3. Increased SR Ca^2+^ Load in NOS3^−/−^ Myocytes with *β*-AR Stimulation

*β*-AR stimulation can result in triggered arrhythmias [36], which are observed in NOS3^−/−^ mice [16]. These premature beats are analogous to afterdepolarizations in isolated myocytes, which we and others have demonstrated to occur in NOS3^−/−^ myocytes [5, 17]. While alterations in K^+^ currents can contribute to the increased incidence of these triggered arrhythmias, we did not observe any difference in K^+^ currents in NOS3^−/−^ myocytes. Another contributing factor to the generation of afterdepolarizations is in increase in the SR Ca^2+^ load [23, 37–39]. Studies have shown that early afterdepolarizations are due to an increase in *I*_Ca_ [40] and delayed afterdepolarizations are due to spontaneous release of Ca^2+^ from the SR (i.e., Ca^2+^ wave) [37]. However, recent data now suggests that early afterdepolarizations caused by *β*-AR stimulation may also be due to spontaneous SR Ca^2+^ release [41, 42]. Thus, an important finding in the present study is the increased SR Ca^2+^ load with *β*-AR stimulation in NOS3^−/−^ myocytes (Figure 4). It is known that enhanced Ca^2+^ influx via I_Ca_ will result in increased SR Ca^2+^ load [33, 43]. Thus, our higher SR Ca^2+^ load in NOS3^−/−^ myocytes is consistent with the observed greater Ca^2+^ influx via *I*_Ca_. In fact, it has been suggested that Ca^2+^ waves and increased *I*_Ca_ act synergistically to produce early and delayed afterdepolarizations [42]. Thus, NOS3^−/−^ myocytes are tailored for afterdepolarizations since these myocytes exhibit an increased *I*_Ca_ and SR Ca^2+^ load during *β*-AR stimulation. In fact, our previous study showed that the vast majority of NOS3^−/−^ myocytes have afterpolarizations [5].

In addition to *I*_Ca_ and the various K^+^ currents, it is known that other currents, most notably the sodium current [44], can not only affect APD but also contribute to the generation of afterdepolarizations. Since the purpose of this study was to examine if NOS3 signaling modulates K^+^ currents, we did not measure sodium current. Thus, the effect of NOS3 on sodium current and its corresponding effect on APD and afterdepolarizations cannot be excluded. Future studies are needed to investigate if NOS3 signaling can modulate the sodium current.

In conclusion, in NOS3^−/−^ mouse ventricular myocytes, *I*_to_, *I*_Ksus_, and *I*_K1_ are normal and do not contribute to the prolonged APD and increased incidence of afterdepolarizations. We suggest that in NOS3^−/−^ myocytes the increase in *β*-AR stimulated *I*_Ca_ is the reason for the prolonged APD. Furthermore, the increased afterdepolarizations in NOS3^−/−^ myocytes during *β*-AR stimulation are caused by the increased *I*_Ca_ along with the increased SR Ca^2+^ load. This may have important implication for arrhythmias and sudden cardiac death in heart failure, where NOS3 expression is decreased [45, 46] and *β*-AR tone is increased [47].
